# Alteration in Community Dynamics of *Chaetoceros curvisetus* and Bacterioplankton Communities in Response to Surfactin Exposure

**DOI:** 10.3390/microorganisms11102596

**Published:** 2023-10-20

**Authors:** Qianwen Shao, Zhujun Zhu, Chengxu Zhou

**Affiliations:** 1 Ningbo Institute of Oceanography, Ningbo 315832, China; zhuzj@nbio.org.cn; 2 Key Laboratory of Marine Ecosystem Dynamics, Second Institute of Oceanography, Ministry of Natural Resources, Hangzhou 310012, China; 3 College of Food and Pharmaceutical Sciences, Ningbo University, Ningbo 315832, China; zhouchengxu@nbu.edu.cn

**Keywords:** surfactin, inhibition effect, algal bloom, bacterioplankton community, size-fractionated filtering

## Abstract

The use of surfactin is a promising method to mitigate algal blooms. However, little is known about surfactin toxicity to algae and bacterioplankton. Here, we treated *Chaetoceros curvisetus*, the dominant species of algal blooms in the East China Sea, with 0, 0.5, 1, 2, 3, and 4 mg/L of surfactin for 96 h to investigate temporal variability. Our results showed that low concentrations of surfactin (<2 mg/L) changed the cell morphology of *C. curvisetus*, and higher concentrations (>3 mg/L) had lethal effects. Meanwhile, we examined the community dynamics of the free-living (FL, 0.22–5 μm) and particle-attached (PA, >5 μm) bacterioplankton of *C. curvisetus* in response to different surfactin concentrations and cultivation periods. Both PA and FL bacterioplankton were mainly composed of Proteobacteria, Actinobacteria, and Bacteroidetes, while FL bacterioplankton were more diverse than PA bacterioplankton. The variations of FL and PA bacterioplankton were significantly constrained by the surfactin concentration. Surfactin changed the lifestyle of some bacterioplankton from FL to PA, which mainly belonged to abundant bacterioplankton. Furthermore, we identified some surfactin-sensitive species/taxa. Our study will help enhance the ability to predict marine microbial responses under the effect of surfactin, providing a research foundation for this new harmful algal bloom mitigation method.

## 1. Introduction

Harmful algal blooms (HABs) represent a broad suite of phytoplankton, macroalgae, and cyanobacteria that can have significant impacts on ecological resources, human health, and coastal economies [1]. Research on the development of techniques to control or mitigate algal blooms is a promising area. Methods of controlling HABs can be separated into three categories based on their mode of action, including physical mitigation methods (e.g., clay flocculation), chemical (e.g., copper sulphate and ozone), and biological (e.g., algicidal bacteria) control methods [2]. Although several of these methods show promise, the direct control of algal blooms is a sensitive issue because there is always the risk of negatively impacting other parts of the aquatic ecosystem.

Recent studies indicate the potential of a group of substances known as biosurfactants as potential agents to manage algal blooms [3,4]. The greatest advantage of biosurfactants when compared with synthetic surfactants is they are easily biodegraded, making them environmentally acceptable in contrast to some synthetic algicides [5]. As most of the biosurfactant research to date has been performed with rhamnolipids, other biosurfactants need to be investigated as they may have more promising properties. Surfactin, a bacterial cyclic lipopeptide, is produced by various strains of *Bacillus subtilis* [6] and is primarily recognized as one of the most effective biosurfactants. Surfactin is named for its exceptional surfactant activity [7]. It lowers the surface tension of water from 72 to 27 mN/m at a concentration as low as 0.005% and is a very powerful biosurfactant [8]. Surfactin possesses a number of biological activities such as the ability to lyse bacterial spheroplasts and protoplasts, and inhibit cyclic 3,5-monophosphate diesterase [9]. Considering the deleterious effect of harmful algal blooms on the environment and the opportunity to avoid the secondary pollution problem encountered with many other control strategies, the use of surfactin is a promising method to mitigate algal blooms.

Previous studies on biosurfactants as potential HABs mitigation agents have focused on the marine dinoflagellates, including *Alexandrium tamarense*, *Alexandrium minutum*, *Karenia brevis*, *Heterosigma akashiwo*, *Cochlodinium polykrikoides*, *Gymnodinium* sp., and *Prorocentrum dentatum*. These species were all observed to be sensitive, although to different extents, to the glycolipid biosurfactants sophorolipid and rhamnolipid [4,10,11]. However, we have little knowledge about the algicidal effects of surfactin. Although Ahn et al. (2003) [3] revealed that the culture broth of *Bacillus subtilis* C1 containing surfactin at 10 mg/L effectively suppressed cyanobacterial growth and speculated that the damage to *Microcystis aeruginosa* was mainly due to the destabilization of the membranes by the surfactin, they did not isolate the biosurfactant from the culture broth to ensure its algicidal activity or elucidate the mechanism further.

In recent years, *Chaetoceros curvisetus* blooms have been recorded in the East China Sea [12], Andaman Islands [13], west Scotland [14], and other areas. The centric diatom *Chaetoceros* sp. is regarded as harmful algae although it does not secrete any harmful toxins. The setae of the diatom are easily broken, and if large quantities lodge in the gills of a fish, they may kill the fish [15]. The secondary spines anchor the setae to the sensitive gill tissue, causing irritation, and the fish reacts by producing mucus. Eventually, it dies from suffocation. Fish mortalities due to this species have already been reported in aquaculture farms [14] and pen cultures [16]. Therefore, there is a need for more study on the short-term ecotoxicological effects of surfactin on the bloom-forming diatom *C. curvisetus*. Determining the temporal variability of *C. curvisetus* could provide a clue as to how diatom communities respond to surfactin.

Bacterioplankton play crucial roles in biogeochemical cycling in marine ecosystems [17] and the biodegradation of organic compounds [18]. Thus, bacterioplankton could be promising targets used to evaluate the ecological effect of contamination at the community level in coastal waters. Bacterioplankton can be classified into two types of communities depending on their relationship with the particulate matter in the water (e.g., free-living (FL) and particle-attached (PA) lifestyles) [19,20]. FL and PA bacterioplankton have been regarded as interacting assemblages [21]. Increasing evidence indicates that the exchange or transition of bacterial lifestyles is influenced by chemical triggers, substrate availability, turbulence, and eutrophication [21,22]. Hence, there is a need for gaining a more in-depth understanding of the transition between these two types of bacterial lifestyles in response to surfactin. Moreover, we wanted to find some surfactin-sensitive species/taxa, which can be used in the automated monitoring instruments for bacterioplankton in situ to help clarify the influence of surfactin on the coastal marine ecosystem. Microbial communities in natural ecosystems are typically composed of a small number of abundant bacterioplankton and a far greater number of rare bacterioplankton [23]. Abundant bacterioplankton dominate major ecosystem processes (for example, carbon flow and nutrient cycling), whereas rare bacterioplankton can be regarded as propagule banks and play minor but non-negligible roles [24]. As such, we further divided bacterioplankton into abundant/rare bacterioplankton to find surfactin-sensitive species/taxa.

The use of surfactin has not been fully demonstrated in coastal environments [3]. Its effectiveness in mitigating algal bloom impacts remains an open question, and the mechanisms of surfactin toxicity to marine bacterioplankton are still not well resolved. Therefore, all of these questions need further and more in-depth investigation to provide a foundation for this proposed new HABs mitigation method. In this study, we aim (i) to investigate the algicidal effect of surfactin on *C. curvisetus*, which has not been tested previously; (ii) to provide a glimpse into the varying trend of the PA and FL bacterioplankton in response to the surfactin concentration and cultivation period using 16S rRNA gene amplicon sequencing; and (iii) to find surfactin-sensitive species/taxa.

## 2. Materials and Methods

### 2.1. Algal Cultures

*C. curvisetus* was originally isolated from the Changjiang estuary, East China Sea. The stock culture maintenance and the experiments were conducted in the Algal Culture Laboratory, Ningbo University. The culture of *C. curvisetus* was grown at 22 ± 1 °C, and approximately 54 μmol photons m^−2^ s^−1^ with a photoperiod cycle of 12:12 h L:D (light:dark). The *C. curvisetus* culture was maintained in a conical flask filled with 4 L of f/2 medium nutrients [25] with the addition of silica, prepared using East China Sea water adjusted to a salinity of 24 by the addition of sterile double-distilled water. The cells used in the experiment were in the exponential phase of the growth curve.

### 2.2. Experimental Design

For each algal culture, aliquots of 100 mL culture were transferred to a total of 28 transparent conical flasks, containing four replicates of one control group (pure seawater) and six surfactin treatments (0, 0.5, 1, 2, 3, and 4 mg/L surfactin). Six surfactin treatments were spiked with ethanol as a carrier solvent for surfactin, which contained the same amount of ethanol (0.04 *v*/*v* %) to make these systems have the same initial background. Notably, 0 mg/L surfactin treatment contained 0.04 *v*/*v* % ethanol, which was different from the control group (pure seawater). Finally, the effect of the surfactin on the abundance of *C. curvisetus* was studied using an inverted light microscope (Leica DM500, Heerbrugg, Switzerland) at 400× magnification. Changes in the morphology of the algal cells were also noted after 24, 48, 72, and 96 h for the different treatments.

After microscopic examination, each experimental sample was immediately filtered through 5-μm and 0.22-μm pore-size membranes (47 mm diameter polycarbonate, Millipore, Burlington, MA, USA) to collect FL and PA bacterioplankton. Bacterioplankton samples were divided into two size fractions: 0.22–5 μm (FL) and >5 μm (PA) to operationally separate free-living cells from those attached to *C. curvisetus*. The filters were then stored at −80 °C until DNA extraction. To prevent contamination between samples, the filtration systems were carefully washed with sterile water before bacterioplankton collection and filtration.

### 2.3. DNA Extraction, PCR Amplification, and Illumina Miseq Pyrosequencing

DNA extraction was performed using the Power Soil DNA isolation kit (Mo Bio, Carlsbad, CA, USA), according to the manufacturer’s instructions. The V3–V4 region of the bacterial 16S rDNA gene was amplified using primer set 341F (5′-CCTACGGGNGGCWGCAG-3′) and 805R (5′-GACTACHVGGGTATCTAATCC-3′). PCR amplifications were performed under the following procedures: 94 °C for 3 min, 25 cycles of denaturation at 94 °C for 30 s, annealing at 50 °C for 20 s, and extension at 72 °C for 30 s, and finalized with a 10-min extension step at 72 °C. Then, PCR products were purified using a PCR fragment purification kit (Takara, Tokyo, Japan) and quantified using a Quant-It Pico Green kit (Invitrogen, Carlsbad, CA, USA) with a Qubit fluorometer (Invitrogen). High-throughput gene sequencing was then performed on a MiSeq platform (Illumina, San Diego, CA, USA) in a paired-end 300-bp sequence read run at Sangon Biotech Co. Ltd. (Shanghai, China).

### 2.4. Processing of Pyrosequencing Data

The QIIME v 1.8 [26] and USEARCH v 6.1 [27] pipelines were used to perform quality control and to cluster sequences into operational taxonomic units (OTUs). Paired-end reads were merged with fast-length adjustment of short reads (FLASH) [28]. Raw sequences were demultiplexed and filtered to remove low-quality sequences using QIIME. Chimeras were detected and removed with USEARCH using the SILVA 128 database [29]. The remaining sequences were clustered into OTUs using a 97% sequence identity cutoff with UCLUST [30]. The taxonomic classifications were carried out using the SILVA 128 reference database. OTUs affiliated with chloroplast, mitochondrion, unclassified sequences, and singletons were removed. After these procedures, the number of final high-quality clean reads of bacterioplankton in each sample ranged from 10,819 to 94,695 reads, yielding a total of 2113 OTUs (Table A1).

### 2.5. Statistical Analysis

All statistical analyses were performed in R v3.6.1 software. Prior to statistical analyses, all biological data were Hellinger-transformed to improve normality and homoscedasticity [31].

#### 2.5.1. Bacterioplankton Community Composition Analyses

The bacterioplankton community compositions were analyzed at the phylum and class levels (top seven in relative abundance), and visualized using the barplot function in R. Alpha diversity indices (Shannon–Wiener, Simpson, Evenness, and Chao1 indices) were calculated from the OTU dataset in QIIME [26]. OTUs were classified into two categories: abundant OTUs and rare OTUs. Referring to a previous study [32,33], abundant OTUs were defined as those with a representation ≥1% within a sample, but never rare (<0.01%), and rare OTUs were defined as having an abundance <0.01% within a sample, but never ≥1%, based on all samples. In addition, sensitive OTUs, which were detected at different cultivation periods, were identified when their relative abundances during one period changed more than 10 or less than 0.1 times compared with other periods. The dynamics of the occurrences and relative abundances of sensitive OTUs were visualized with Heatmap, using the “pheatmap” package in the R environment.

One-way analysis of variance (ANOVA) was applied to investigate the impacts of size fraction, cultivation period, changes in algal cell, and surfactin concentration on the OTU number and bacterial alpha diversity indices. Changes in algal cell were defined as the change in cell concentration of the algal population over the last 24 h of culture. Non-metric multidimensional scaling (NMDS) using a Bray−Curtis metric was applied to phylogenetically visualize the overall variation in bacterial community composition. A measure of goodness of fit of the ordination was given by a stress value, which was set at <0.20 to minimize misinterpretation [34]. Permutational multivariate analysis of variance (PERMANOVA) was applied to partition the variation in bacterial community composition using the “vegan” package [31].

#### 2.5.2. Particle-Association Niche Index

To numerically characterize each OTU in relation to its occurrence and relative abundance in the PA and FL samples, we employed a particle-association niche index (PAN index). This index served as a measure of the position of an OTU in a continuous niche space ranging from a completely FL to a completely PA lifestyle. Only the OTUs with >10 sequences were used to calculate the PAN index. The PAN index was computed by an abundance-weighted mean: for a given OTU, we recorded its abundance in every sample and recorded the size fraction to which each sample belonged [35,36]. FL and PA bacterioplankton were given a value of 0 and a value of 1, respectively. Thus, an OTU occurring only in PA samples would have a PAN index value of 1 and an OTU strictly occurring in FL samples would have a value of 0. An OTU equally distributed across FL and PA samples would have a PAN index value of 0.5. Using this index, each OTU can be positioned in a continuum describing its lifestyle preference.

## 3. Results

### 3.1. Effect of Surfactin on C. curvisetus

**Figure 1:**
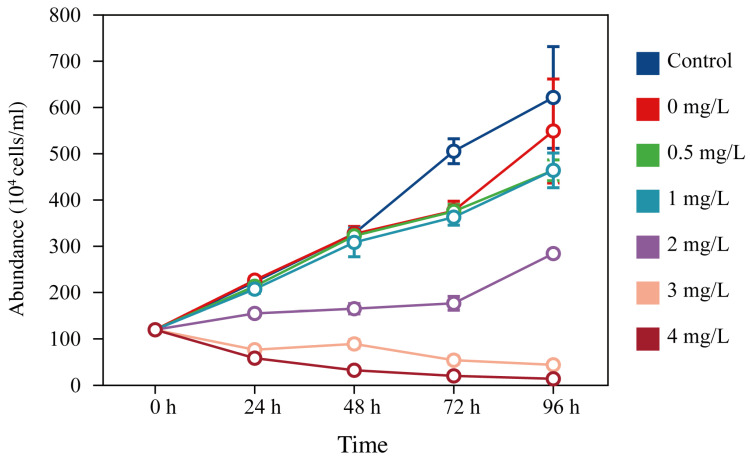
The culture abundance (10^4^ cells/mL, mean ± SD) of *Chaetoceros curvisetus* in the control group (pure seawater) and six surfactin treatments (0, 0.5, 1, 2, 3, and 4 mg/L surfactin) throughout a 96-h cultivation period. Data are expressed as means ± standard error (error bars).

Surfactin showed growth-inhibiting effects on the *C. curvisetus* at all tested concentrations, and the inhibitory effects increased with the increasing concentration of the surfactin (Figure 1). Compared to the cells in the control, the cell abundance of the algae decreased slightly on the second and third day after the addition of the surfactin at 0 mg/L, 0.5 mg/L, and 1 mg/L, and decreased significantly after 72 h. Treatments with 2 mg/L of surfactin led to significant suppression for *C. curvisetus* growth; the algal cell abundance was significantly less than 1 mg/L, and most algal cells were transformed from multicellular chains into single cells after 24 h. When the concentration was further increased to 3 mg/L and 4 mg/L, the surfactin showed a substantial killing effect on *C. curvisetus*; almost all lysed and disappeared within 48 h.

### 3.2. Bacterioplankton Community Composition

**Figure 2:**
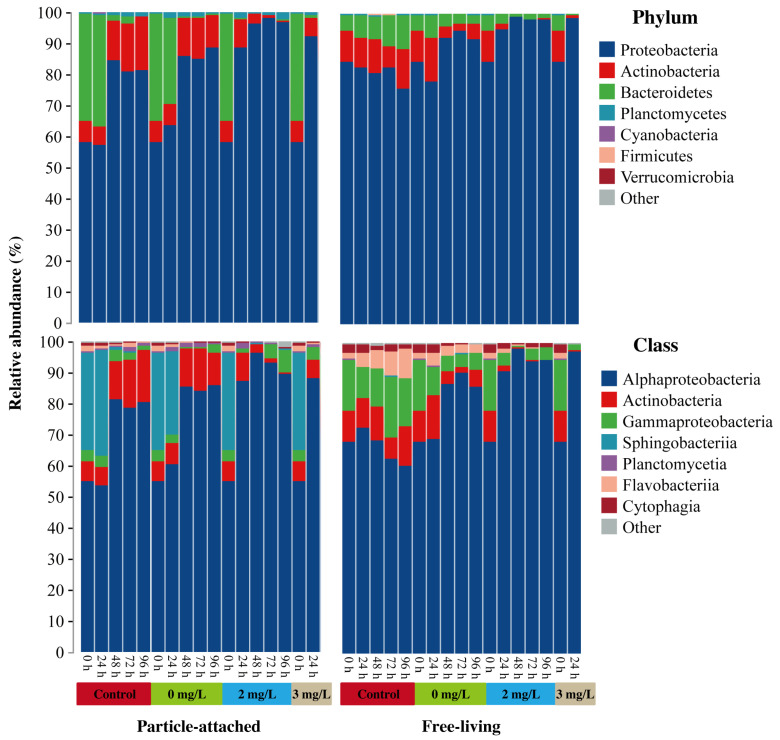
Relative abundance of particle-attached and free-living bacterioplankton samples at the phylum and class levels. Control group (pure seawater) and two surfactin treatments (0 and 2 mg/L surfactin) were analyzed throughout a 96-h cultivation period, while 3 mg/L of the surfactin treatment was analyzed throughout a 24-h cultivation period.

Approximately 99.67% of the obtained sequences could be classified at the phylum level. In the PA bacterioplankton, Proteobacteria (dominated by the Alphaproteobacteria and Gammaproteobacteria classes) was generally the most dominant phylum, with the relative abundance ranging from 57.41% to 98.10% (Figure 2), followed by Actinobacteria (from 0.66% to 16.91%, composed mainly of the Actinobacteria class), Bacteroidetes (from 0.05% to 35.76%, composed mainly of the Sphingobacteriia class), and Planctomycetes (from 0.49% to 2.18%, dominated by the Planctomycetia and Phycisphaerae classes), which cumulatively accounted for more than 99% of the bacterial sequences. In the FL bacterioplankton, Proteobacteria (dominated by the Alphaproteobacteria and Gammaproteobacteria classes) was also the most dominant phylum with the relative abundance ranging from 76.03% to 98.94% (Figure 2), followed by Actinobacteria (from 0.20% to 13.97%, mainly composed of the Actinobacteria class), Bacteroidetes (from 0.37% to 11.06%, dominated by the Flavobacteriia and Cytophagia classes), and Firmicutes (from 0.01% to 0.64%, mainly composed of the Clostridia class), which cumulatively accounted for more than 99% of the bacterial sequences.

The cultivation period and surfactin concentration also resulted in substantial changes in the relative abundances of the dominant taxa (Figure 2). In the PA bacterioplankton, the relative abundance of Sphingobacteriia reached 26.37−34.19% in the first 24 h and decreased to 0.05−0.84% later in the control and 0 mg/L surfactin samples, while the relative abundances of Alphaproteobacteria and Actinobacteria increased from 53.50−60.47% to 78.69−85.87%, and from 5.96−6.66% to 10.27−16.91%, respectively. In the treatments with 2 and 3 mg/L of surfactin, the relative abundance of Sphingobacteriia decreased from 31.58% at 0 h to near zero later, while the relative abundance of Alphaproteobacteria increased from 54.82% at 0 h to more than 87% later. In the FL bacterioplankton, the cultivation period had little effect on the bacterioplankton community composition in the control samples. The addition of ethanol and surfactin showed a negative effect on taxonomic composition, leading to marked decreases in the relative abundances of Gammaproteobacteria and Actinobacteria, and the increase in the relative abundance of Alphaproteobacteria.

### 3.3. General Patterns of Bacterioplankton Alpha Diversity

**Figure A1:**
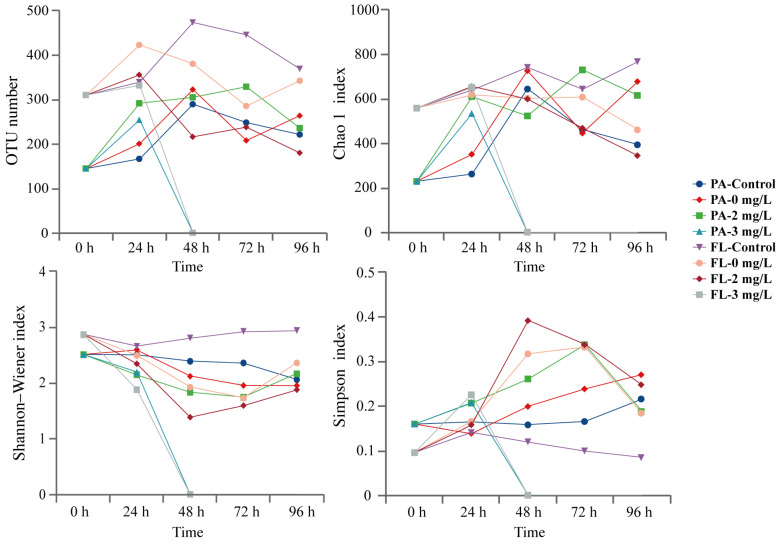
α-diversity indices changes of particle-attached (PA) and free-living (FL) bacterioplankton in control group (pure seawater) and three surfactin treatments (0, 2 and 3 mg/L surfactin) throughout a 96-h cultivation period (0 h, 24 h, 48 h, 72 h, 96 h), including OTU number, Chao 1, Shannon–Wiener and Simpson index.

The observed total number of bacterial OTUs was 2113 in this study. In the comparison between size fractions, the OTU number of FL bacterioplankton (mean ± s.e., 324.00 ± 79.09) was significantly higher than that in PA bacterioplankton (240.43 ± 52.52) (*p* < 0.01), whereas the Chao1, Shannon–Wiener, and Simpson indices showed no significant difference (*p* > 0.05) (Table 1). For the values, the Chao1 (PA: 514.98 ± 157.90; FL: 597.15 ± 107.31), Shannon–Wiener (PA: 2.18 ± 0.25; FL: 2.27 ± 0.51), and Simpson (PA: 0.20 ± 0.05; FL: 0.21 ± 0.10) indices of FL bacterioplankton were higher than the PA bacterioplankton (Figure A1). In addition, the Shannon–Wiener indices of all and PA bacterioplankton were significantly affected by the changes in algal cell, and PA bacterioplankton was also significantly affected by the different cultivation periods (*p* < 0.05). Moreover, the Shannon–Wiener and Simpson indices of all and FL bacterioplankton were significantly affected by the different surfactin concentrations (*p* < 0.05) (Table 1).

### 3.4. Abundant, Rare, and Sensitive OTUs

The PA bacterioplankton was made up of 1281 OTUs, and FL bacterioplankton was comprised of 1651 OTUs. Among the 2113 OTUs observed in all samples, 13 OTUs were abundant OTUs, which accounted for 83.29% of the total sequences, and 2063 OTUs were rare OTUs, which accounted for 1.60% of the total sequences.

**Figure 3:**
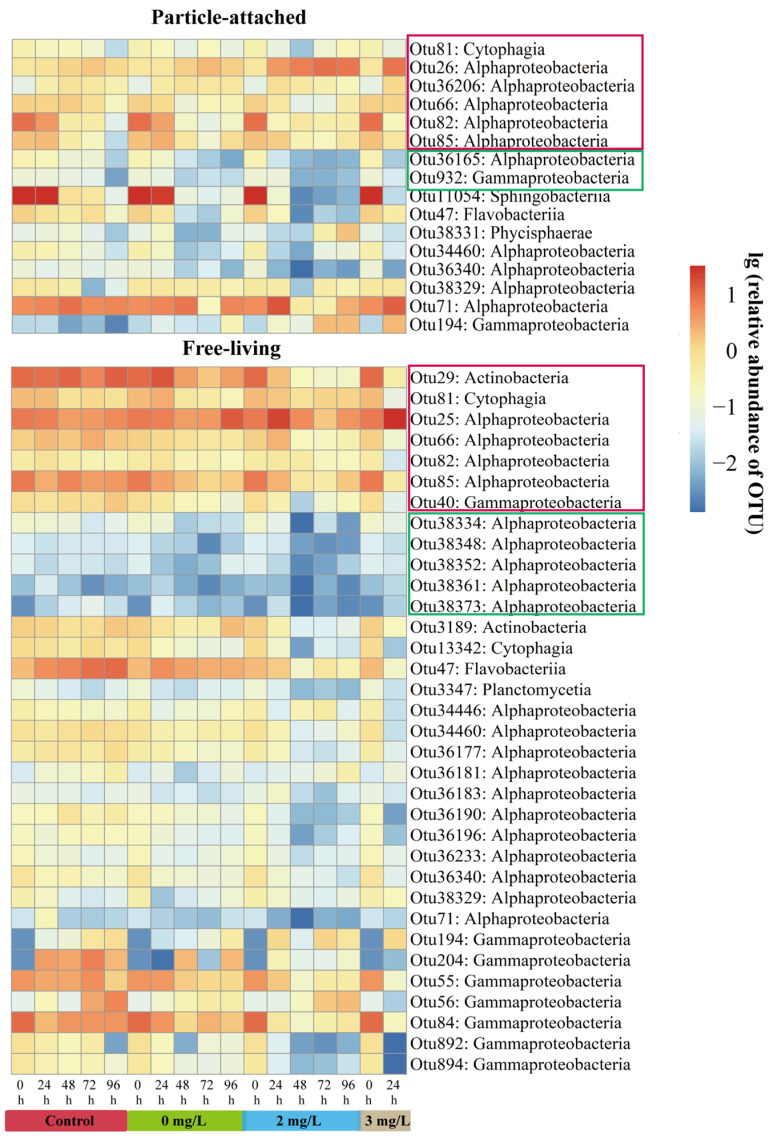
Heatmap of the relative abundance of sensitive operational taxonomic units (OTUs) at the class levels in the particle-attached and free-living bacterioplankton samples after lg transformation. Abundant OTUs are marked in the red boxes, while rare OTUs are marked in the green boxes.

The heatmap plot illustrates the temporal pattern in distribution of sensitive OTUs (Figure 3). Only 16 sensitive OTUs were detected in the PA samples containing 6 abundant OTUs (Cytophagia and Alphaproteobacteria classes) and 2 rare OTUs (Alphaproteobacteria and Gammaproteobacteria classes), while 34 sensitive OTUs were found in the FL samples containing 7 abundant OTUs (Actinobacteria, Cytophagia, Alphaproteobacteria, and Gammaproteobacteria classes) and 5 rare OTUs (Alphaproteobacteria class) (for detailed species annotation information, see Table A2). Most abundant and rare OTUs detected as the sensitive OTUs had the highest relative abundance at 0 h, which then decreased as the surfactin concentration and cultivation time increased.

### 3.5. Effect of Size Fraction, Cultivation Period, Changes in Algal Cell, and Surfactin Concentration on the Bacterioplankton Community

**Figure 4:**
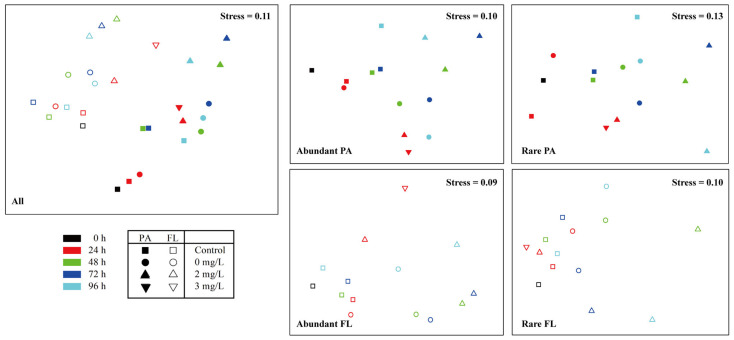
Nonmetric multidimensional scaling (NMDS) plots based on the Bray–Curtis dissimilarity of bacterioplankton community composition. All represents the whole bacterioplankton community including both particle-attached (PA) and free-living (FL) bacterioplankton. Abundant indicates abundant taxa, while rare indicates rare taxa. Different colors represent different cultivation periods (0 h, 24 h, 48 h, 72 h, 96 h). Different shapes represent control group (pure seawater) and three surfactin treatments (0, 2 and 3 mg/L surfactin), while solid shapes indicate PA bacterioplankton, and hollow shapes indicate FL bacterioplankton.

To highlight the successional process of community dynamics, we obtained five NMDS ordination plots for the bacterioplankton community (Figure 4). All two-dimensional NMDS had stress values lower than 0.20, indicating that these ordination patterns were acceptable and bacterioplankton communities showed a distinct successional process in composition according to bacterial size fraction, cultivation period, and surfactin concentration. Furthermore, based on the OTUs detected across the samples, a PERMANOVA was performed to quantitatively evaluate the effects of bacterial cultivation period, changes in algal cell, surfactin concentration, and size fraction on the Bray–Curtis dissimilarity of community composition. The results showed that bacterioplankton community composition was significantly constrained by the size fraction (global R = 0.238, *p* = 0.001) and surfactin concentration (global R = 0.138, *p* = 0.001) on the whole (Table 2). Furthermore, surfactin concentration showed significant explanatory power to the variation of all, abundant, and rare PA bacterioplankton as well as all and abundant FL bacterioplankton, while cultivation period was only significantly associated with abundant and rare PA bacterioplankton, and changes in algal cells were only significantly associated with rare PA bacterioplankton.

### 3.6. Lifestyle Transition of Bacterioplankton

**Figure 5:**
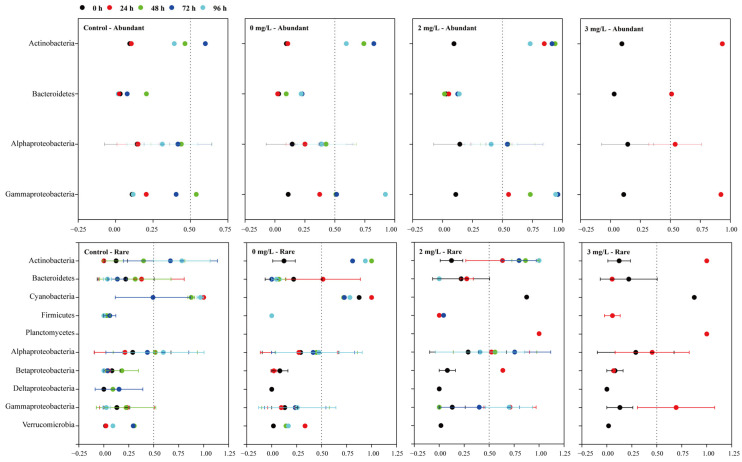
Particle-association niche (PAN) index of the abundant and rare bacterial taxa at the phylum/class level in the control group (pure seawater) and three surfactin treatments (0, 2 and 3 mg/L surfactin). The vertical dashed line corresponds to a 0.5 PAN index, which indicates that the relative abundance of free-living bacterioplankton is equal to that of particle-attached bacterioplankton. Error bars indicate standard errors of the PAN index of operational taxonomic units (OTUs) belonging to each phylum/class. Data are expressed as means ± standard error (error bars).

Bacterioplankton showed a transition or switching phenomenon between the two different lifestyles at the OTU and taxonomic levels. Overall, the majority of phyla/classes in the biosphere had a mean PAN index < 0.5, supporting a preference for the FL lifestyle during the sampling period (Figure 5). All abundant OTUs showed a strong transition from FL to PA lifestyle following the cultivation of *C. curvisetus*, especially for Actinobacteria and Gammaproteobacteria. For the rare OTUs, Actinobacteria, Alphaproteobacteria, and Gammaproteobacteria showed a strong transition from FL to PA lifestyle. Bacteroidetes, Firmicutes, Betaproteobacteria, Deltaproteobacteria, and Verrucomicrobia tended to be more FL with the extension of cultivation time, while Cyanobacteria and Planctomycete preferred PA lifestyle.

## 4. Discussion

### 4.1. Inhibition Effect of Surfactin on Harmful Algae

The control of HABs means the early removal of the overpopulation within a relatively short time. In the present study, low concentrations of surfactin reduced the efficiency of the motility in *C. curvisetus*. When the surfactin concentration was greater than 3.0 mg/L, the population of *C. curvisetus* in the logarithmic phase was quickly destroyed and completely lysed within 48 h. Furthermore, ethanol is widely used as a carrier for a wide spectrum of medicines [37], but the effect of ethanol (carrier solvent) on *C. curvisetus* as a whole is largely unknown. Compared with the control group (pure seawater) and 0 mg/L surfactin treatment (seawater with 0.04 *v*/*v* % ethanol), there was almost no difference in algae abundance between two groups in the first 48 h, and algae abundance of 0 mg/L surfactin treatment decreased in the last 48 h (Figure 1), which means that the addition of ethanol may gradually lyse susceptible phytoplanktonic cells with extending cultivation time. Although ethanol can serve as a carbon source for cultivation of microalgae strains, a previous study reported that ethanol even at a concentration as low as 0.05 *v*/*v* %, could exert inhibition on *Chlorella vulgaris* and *Selenastrum capricornutum* growth [38], which was similar to our results.

Biosurfactants have potential for the selective control of harmful algae. For example, Gustafsson et al. (2009) [11] reported that 50 mg/L of the rhamnolipid was needed to kill diatom species *Pseudonitzschia* sp., while both Wang et al. (2005) [4] and Gustafsson et al. (2009) [11] elucidated that a concentration of 5 mg/L rhamnolipid was enough to cause a 100% algicidal effect on marine dinoflagellate species. Compared to the rhamnolipid experiments, Gong et al. (2006) [39] showed that the growth of *C. curvisetus* was strongly inhibited in a medium containing rhamnolipid (>7 mg/L), which suggested that surfactin (>3 mg/L) was more efficient than rhamnolipid at algae lysing. The primary site for cellular damage caused by biosurfactants is on the cytoplasmic membranes [40]. The different structures of biosurfactants confer different properties, including cell toxicity by interfering with cell membrane permeability. A likely explanation for the algicidal effect is that surfactin can inhibit fibrin clot formation [6], induce the formation of ion channels in lipid bilayer membranes [41], and block the activity of cyclic adenosine monophosphate [42], which eventually changes the rigidity of the cell membrane. The cell wall also plays a role in maintaining the membrane integrity [10] and different cell wall structures of the organisms were responsible for different effects of biosurfactants. For example, the protein and lipopolysaccharide moieties of the cell wall of Gram-negative bacteria could protect the cell membrane from surfactant attack [43].

### 4.2. Effects of Surfactin on Bacterioplankton Communities

Generally, the PA microbial communities have relatively low abundance and diversity compared with the FL microbial communities [44], which is consistent with our results (Figure A1). The PA fraction is relatively enriched in members of Gammaproteobacteria, Verrucomicrobia, Bacteroidetes, Firmicutes, and Planctomycetes [45,46], while the FL assemblages are often populated by members of Alphaproteobacteria and Deferribacteres [47,48]. A high proportion of overlap of the microbial compositions between PA and FL fractions implies that most microorganisms are potentially generalists, with dual PA and FL lifestyles for versatile metabolic flexibility [47]. In this study, Alphaproteobacteria, Gammaproteobacteria, Actinobacteria, and Bacteroidetes were the major overlapping phylum and classes in both the PA and FL microbial fractions (Figure 2). This pattern has been confirmed to be closely related to marine diatom blooms [49,50].

This preliminary study was intended to provide a glimpse into the varying trend of the bacterioplankton community in response to surfactin, and temporal variability did not conceal the surfactin-induced patterns. The concentrations of surfactin of more than 2 mg/L rapidly affected the bacterioplankton community composition (Figure 2), as observed in bacterial alpha diversity (Figure A1). Different degrees of response by FL and PA bacterioplankton communities to the surfactin concentration changes were found during the growth of *C. curvisetus*. The FL bacterioplankton community was more significantly related to the different concentrations of surfactin both in the diversity indices and community composition (Table 1 and Table 2), while the PA bacterioplankton community was quite conserved. Some studies have found that FL bacterioplankton appear to be more sensitive to environmental variables than PA bacterioplankton [51], which is similar to our results. Some studies have suggested that PA bacterioplankton communities may be more sensitive to global change stressors, including land use change, species invasions, and changes in geochemical cycles [52]. Recently, Tang et al. (2017) [22] found that environmental factors affected bacterial communities in much the same way regardless of bacterial lifestyles in Lake Taihu, China. 

We first used the PAN index to quantitatively estimate the lifestyle alternation between FL and PA bacterioplankton at the phylum/class level in response to different concentrations of surfactin and the growth of the *C. curvisetus*. Despite differences between the FL and PA bacterial community composition (Figure 2 and Table 2), we found strong evidence of bacterial lifestyle shifts from FL to the PA states, especially for all abundant bacterioplankton and some rare bacterioplankton belonging to Actinobacteria, Alphaproteobacteria, and Gammaproteobacteria (Figure 5). This possible lifestyle transition has been detected through community composition, the production of exoenzymes, or community transcription patterns in marine environmental mesocosms [53] or in experimental mesocosms [54]. One possible explanation is that most of the marine bacterioplankton are generalists with dual-lifestyle strategies [55,56]. The other explanation is that bacterial populations may switch between FL and PA lifestyles depending on substrate availability and surrounding chemical triggers [21,57], such as the changes of surfactin concentration and *C. curvisetus* abundance in our study. Compared with FL bacterioplankton, PA bacterioplankton had several advantages in hydrolysis or the decomposition of marine organic matter, biomass production, and carbon cycling [58,59]. First, many of the PA bacterioplankton can be part of biofilms or zoogloeae. The PA lifestyle exhibits many advantages compared to that of FL planktonic bacteria, including protection from predators and many other environmental changes [60]. Second, the dynamics of PA bacterioplankton were more closely related to the growth of the *C. curvisetus*, compared to FL bacterioplankton (Table 1 and Table 2). Surfactin lysed algal cells and may increase bacterial adhesion surface area. The PA bacterioplankton have chemotaxis and motility, which allow for the coupling between PA bacterioplankton and phytoplankton, with bacteria gathering within the dissolved organic carbon (DOC)-rich “phycosphere” surrounding individual algal cells [61]. Finally, PA bacterioplankton generally have large and variable genomes that contain genes enabling a variety of metabolic capabilities, which are thought to equip cells to take advantage of patches of organic matter and adapt rapidly to a changing environment [62].

### 4.3. Sensitive Bacterial Taxa Associated with Surfactin on a Temporal Scale

The exploration of microbial taxa associated with surfactin could provide potential bioindicators. A comprehensive study suggested that if a certain taxon could be consistently and repeatedly detected at great abundance in a given location, there would be a high possibility that this taxon is environment-specific rather than a transient passer-by [63]. We extend this concept to propose that if a taxon shows frequent and stable correlation with the concentration of surfactin, it is possible that this taxon plays an important ecological role in response to surfactin exposure. In our study, there were six abundant OTUs and two rare OTUs in the PA-sensitive OTUs, while the FL-sensitive OTUs contained seven abundant OTUs and five rare OTUs (Figure 3). Abundant bacterioplankton had greater variation than rare bacterioplankton due to the different cultivation periods and surfactin concentrations (Figure 3 and Table 2), which may have been due to rare phylotypes (species) being protected from active loss because of their low abundance [64].

Sensitive bacterial taxa in our study belonged to the Actinobacteria, Bacteroidetes, Proteobacteria, and Planctomycetes phyla (Table A2). The rapid change in the abundance of sensitive bacterial taxa involves the properties of these bacterioplankton. One explanation is that sensitive bacterial taxa have fast generation times and short-lived population maxima [35]. For example, Alphaproteobacteria, especially members of the Rhodobacterales clade (in particular, OTUs 25 and 26, which were the abundant OTUs in the FL and PA samples, respectively) and the Rhizobiales clade (in particular, OTU 36206, which was an abundant OTU in the PA samples), were capable of rapidly changing their metabolic functioning in response to changing conditions, thus enhancing their competitive ability [65]. The other explanation is that sensitive bacterial taxa can be seen as fast-growing r-strategists that specialize in the initial attack of highly complex organic matter [49], such as surfactin. For example, Gammaproteobacteria, especially members of the Alteromonas–Pseudomonas–Vibrio group (especially OTU 194), are frequently characterized as r-strategists [66], colonizing quickly and promoting the growth of more specialized taxa. Carboxylate degradation enzymes were abundant in Gammaproteobacteria, which can degrade allochthonous organic matter [65]. Moreover, some studies have shown that Actinobacteria use lipids in the oceanic environment as a carbon source [67], such as surfactin in our study. Actinobacteria preferred an FL lifestyle at 0 h, and transitioned from FL to PA following the addition of surfactin and the cultivation of *C. curvisetus* (Figure 5). OTU 29 was an abundant OTU in the FL samples that belonged to Actinobacteria phylum and Pontimonas genus (Table A2). Genus Pontimonas species always have small genomes, which are said to be “streamlined” [68]. Streamlined bacteria deal with fewer secondary metabolites, are focused on fewer kinds of nutrient molecules, and tend to be FL, but the addition of surfactin changed their lifestyle. Future studies should explore the species functions of these sensitive bacterial taxa and how they respond to surfactin.

## 5. Conclusions

In this study, we studied surfactin toxicity to *C. curvisetus* and bacterioplankton community composition and examined the community dynamics of the PA and FL bacterioplankton in response to different surfactin concentrations and cultivation periods. However, our assessment was based on laboratory experimental data. Culture experiments could be biased due to the lack of communities from all trophic levels; sediment–water interactions; physical processes such as wind and water renewal, and seasonality; and spatial heterogeneity, thus reducing the complexity in situ [69]. Future investigations based on in situ mesocosms across larger temporal scales, especially for communities with various initial compositions derived from spatial heterogeneity, could be a promising strategy to validate and extend the observed effects of surfactin on the diversity, composition, and lifestyle of marine biota.

## Figures and Tables

**Table 1:** F-values and *p*-values of one-way ANOVA testing the effects of size fraction (SF), cultivation period (CP), changes in algal cell (AC), and surfactin concentration (SC) on the operational taxonomic unit (OTU) number and alpha diversity indices of all, particle-attached (PA), and free-living (FL) bacterial communities.

Factors	All Bacteria (*n* = 28)					PA Bacteria (*n* = 14)				FL Bacteria (*n* = 14)		
	SF	CP	AC	SC		CP	AC	SC		CP	AC	SC
OTU number	10.07 **	0.01	1.15	0.03		1.10	3.15	1.80		0.63	0.05	1.22
Chao 1	2.41	0.45	0.02	0.18		3.04	0.05	3.40		1.36	0.01	3.47
Shannon–Wiener	0.32	2.10	4.39 *	11.46 **		6.03 *	5.57 *	3.00		0.28	3.96	9.13 *
Simpson	0.01	1.90	3.40	11.26 **		3.33	0.91	2.37		0.36	2.34	9.50 **

* *p* < 0.05, ** *p* < 0.01.

**Table 2:** Quantitative effects of size fraction (SF), cultivation period (CP), changes in algal cell (AC), and surfactin concentration (SC) on the variations in bacterioplankton community composition based on permutational multivariate analysis of variance (PERMANOVA). All represents the whole bacterioplankton community including both particle-attached (PA) and free-living (FL) bacterioplankton. Abundant indicates abundant taxa, while rare indicates rare taxa.

PERMANOVA	ALL	PA			FL		
		All	Abundant	Rare	All	Abundant	Rare
Size-fraction	0.238 **	-	-	-	-	-	-
Cultivation period	0.065	0.371	0.228 *	0.127 **	0.277	0.063	0.095
Changes in algal cell	0.04	0.11	0.13	0.19 *	0.07	0.05	0.08
Surfactin concentration	0.138 **	0.320 *	0.339 **	0.109 *	0.445 **	0.359 **	0.098

* *p* < 0.05, ** *p* < 0.01.

## Data Availability

The 16S rRNA gene sequences from this study have been deposited in the public NCBI database under the BioProject accession number PRJNA795844. The BioSample accession numbers are from SAMN30172296 to SAMN30172323.

## Appendix A

**Table A1:** Summary statistics of sequence counts for each bacterioplankton sample before and after quality filtering.

Size-Fraction	Surfactin Concentration	Cultivation Period	Raw Reads	Clean Reads	OTU Numbers
Particle-attached bacterioplankton	Control	0 h	58,626	57,675	140
		24 h	83,029	81,607	161
		48 h	89,206	87,793	280
		72 h	73,336	72,260	240
		96 h	66,999	65,830	214
	0 mg/h	0 h	58,626	57,675	140
		24 h	93,061	91,474	194
		48 h	86,710	85,373	312
		72 h	58,132	57,194	201
		96 h	84,574	83,353	255
	2 mg/h	0 h	58,626	57,675	140
		24 h	80,285	79,119	282
		48 h	94,814	93,409	295
		72 h	114,815	112,780	318
		96 h	69,284	68,173	228
	3 mg/h	0 h	58,626	57,675	140
		24 h	70,615	69,275	246
Free-living bacerioplankton	Control	0 h	70,927	69,692	300
		24 h	81,048	79,802	328
		48 h	85,595	84,288	458
		72 h	96,288	94,789	431
		96 h	73,925	72,435	357
	0 mg/h	0 h	70,927	69,692	300
		24 h	90,509	88,976	409
		48 h	92,050	90,149	368
		72 h	82,260	80,953	276
		96 h	105,669	103,971	331
	2 mg/h	0 h	70,927	69,692	300
		24 h	78,420	77,285	344
		48 h	92,290	90,956	209
		72 h	69,320	68,329	230
		96 h	86,369	85,100	174
	3 mg/h	0 h	70,927	69,692	300
		24 h	76,432	75,244	321

**Table A2:** Summary species annotation of sensitive OTUs in the particle-attached (PA) and free-living (FL) bacterioplankton samples.

Life Mode	Abundance/Rare	OTU	Phylum	Class	Order	Family	Genus
PA/FL	Abundance	Otu81	Bacteroidetes	Cytophagia	Cytophagales	Cyclobacteriaceae	Algoriphagus
PA	Abundance	Otu26	Proteobacteria	Alphaproteobacteria	Rhodobacterales	Rhodobacteraceae	Loktanella
PA	Abundance	Otu36206	Proteobacteria	Alphaproteobacteria	Rhizobiales	Phyllobacteriaceae	unclassified
PA/FL	Abundance	Otu66	Proteobacteria	Alphaproteobacteria	Caulobacterales	Hyphomonadaceae	Oceanicaulis
PA/FL	Abundance	Otu82	Proteobacteria	Alphaproteobacteria	Rhodobacterales	Rhodobacteraceae	unclassified
PA/FL	Abundance	Otu85	Proteobacteria	Alphaproteobacteria	Caulobacterales	Hyphomonadaceae	Maricaulis
PA	Rare	Otu36165	Proteobacteria	Alphaproteobacteria	Rhodobacterales	Rhodobacteraceae	Litoreibacter
PA	Rare	Otu932	Proteobacteria	Gammaproteobacteria	unclassified	unclassified	unclassified
PA	/	Otu11054	Bacteroidetes	Sphingobacteriia	Sphingobacteriales	Saprospiraceae	Phaeodactylibacter
PA	/	Otu47	Bacteroidetes	Flavobacteriia	Flavobacteriales	unclassified	unclassified
PA	/	Otu38331	Planctomycetes	Phycisphaerae	Phycisphaerales	Phycisphaeraceae	Phycisphaera
PA	/	Otu34460	Proteobacteria	Alphaproteobacteria	Rhodospirillales	Rhodospirillaceae	unclassified
PA	/	Otu36340	Proteobacteria	Alphaproteobacteria	Rhodospirillales	Rhodospirillaceae	Oceanibaculum
PA	/	Otu38329	Proteobacteria	Alphaproteobacteria	Rhodobacterales	Rhodobacteraceae	Oceanicola
PA	/	Otu71	Proteobacteria	Alphaproteobacteria	Rhizobiales	Rhizobiales_incertae_sedis	Amorphus
PA	/	Otu194	Proteobacteria	Gammaproteobacteria	Alteromonadales	Alteromonadaceae	Alteromonas
FL	Abundance	Otu29	Actinobacteria	Actinobacteria	Actinomycetales	Microbacteriaceae	Pontimonas
FL	Abundance	Otu25	Proteobacteria	Alphaproteobacteria	Rhodobacterales	Rhodobacteraceae	Oceanicola
FL	Abundance	Otu40	Proteobacteria	Gammaproteobacteria	Gammaproteobacteria_incertae_sedis	Gammaproteobacteria_incertae_sedis_unclassified	Congregibacter
FL	Rare	Otu38334	Proteobacteria	Alphaproteobacteria	Rhizobiales	Bradyrhizobiaceae	Bradyrhizobium
FL	Rare	Otu38348	Proteobacteria	Alphaproteobacteria	Rhizobiales	Phyllobacteriaceae	Hoeflea
FL	Rare	Otu38352	Proteobacteria	Alphaproteobacteria	Rhizobiales	Phyllobacteriaceae	unclassified
FL	Rare	Otu38361	Proteobacteria	Alphaproteobacteria	Rhizobiales	Hyphomicrobiaceae	Hyphomicrobium
FL	Rare	Otu38373	Proteobacteria	Alphaproteobacteria	Rhizobiales	Bradyrhizobiaceae	Bosea
FL	/	Otu3189	Actinobacteria	Actinobacteria	Acidimicrobiales	Acidimicrobiaceae	Ilumatobacter
FL	/	Otu13342	Bacteroidetes	Cytophagia	Cytophagales	Flammeovirgaceae	Fabibacter
FL	/	Otu47	Bacteroidetes	Flavobacteriia	Flavobacteriales	unclassified	unclassified
FL	/	Otu3347	Planctomycetes	Planctomycetia	Planctomycetales	Planctomycetaceae	Rhodopirellula
FL	/	Otu34446	Proteobacteria	Alphaproteobacteria	Rhodobacterales	Rhodobacteraceae	unclassified
FL	/	Otu34460	Proteobacteria	Alphaproteobacteria	Rhodospirillales	Rhodospirillaceae	unclassified
FL	/	Otu36177	Proteobacteria	Alphaproteobacteria	Rhodobacterales	Rhodobacteraceae	unclassified
FL	/	Otu36181	Proteobacteria	Alphaproteobacteria	Rhodobacterales	Rhodobacteraceae	Sulfitobacter
FL	/	Otu36183	Proteobacteria	Alphaproteobacteria	Caulobacterales	Hyphomonadaceae	Glycocaulis
FL	/	Otu36190	Proteobacteria	Alphaproteobacteria	Rhodospirillales	Rhodospirillaceae	unclassified
FL	/	Otu36196	Proteobacteria	Alphaproteobacteria	unclassified	unclassified	unclassified
FL	/	Otu36233	Proteobacteria	Alphaproteobacteria	Rhizobiales	Rhizobiales_incertae_sedis	Amorphus
FL	/	Otu36340	Proteobacteria	Alphaproteobacteria	Rhodospirillales	Rhodospirillaceae	Oceanibaculum
FL	/	Otu38329	Proteobacteria	Alphaproteobacteria	Rhodobacterales	Rhodobacteraceae	Oceanicola
FL	/	Otu71	Proteobacteria	Alphaproteobacteria	Rhizobiales	Rhizobiales_incertae_sedis	Amorphus
FL	/	Otu194	Proteobacteria	Gammaproteobacteria	Alteromonadales	Alteromonadaceae	Alteromonas
FL	/	Otu204	Proteobacteria	Gammaproteobacteria	Gammaproteobacteria_incertae_sedis	Gammaproteobacteria_incertae_sedis_unclassified	Pseudohaliea
FL	/	Otu55	Proteobacteria	Gammaproteobacteria	Alteromonadales	Alteromonadaceae	Aliiglaciecola
FL	/	Otu56	Proteobacteria	Gammaproteobacteria	Alteromonadales	Alteromonadaceae	Marinobacter
FL	/	Otu84	Proteobacteria	Gammaproteobacteria	Gammaproteobacteria_incertae_sedis	Gammaproteobacteria_incertae_sedis_unclassified	Pseudohaliea
FL	/	Otu892	Proteobacteria	Gammaproteobacteria	Oceanospirillales	unclassified	unclassified
FL	/	Otu894	Proteobacteria	Gammaproteobacteria	unclassified	unclassified	unclassified
